# The Diagnostic Utility of Volatile Organic Compounds in Inflammatory Bowel Disease: A Systematic Review and Meta-Analysis

**DOI:** 10.1093/ecco-jcc/jjad132

**Published:** 2023-08-04

**Authors:** Ashwin Krishnamoorthy, Subashini Chandrapalan, Marriam Ahmed, Ramesh P Arasaradnam

**Affiliations:** Warwick Medical School, University of Warwick, Coventry, UK; Department of Gastroenterology, Epsom and St Helier University Hospitals NHS Trust, Carshalton, Surrey, UK; Department of Surgery University Hospital Coventry and Warwickshire, Coventry, UK; Department of Gastroenterology, University Hospital Coventry and Warwickshire, Coventry, UK

**Keywords:** Inflammatory bowel disease, volatile organic compounds, biomarkers

## Abstract

**Background:**

Volatile organic compounds [VOCs] show promise as potential biomarkers of for ulcerative colitis and Crohn’s disease, two chronic, idiopathic, gastrointestinal disorders with diagnostic and management challenges. Non-invasive biomarkers aid early diagnosis and management. In this study we review studies of diagnostic accuracy of VOCs in inflammatory bowel disease.

**Methods:**

A systematic search was carried out on the Pubmed and Scopus databases; with 16 studies reviewed and meta-analysis carried out on 10.

**Results:**

Meta-analysis of 696 inflammatory bowel disease [IBD] cases against 605 controls revealed a pooled sensitivity and specificity of 87% (95% confidence interval [CI], 0.79 - 0.92) and 83% [95% CI, 0.73 - 0.90], respectively. Area under the curve [AUC] was 0.92.

**Conclusion:**

VOCs perform very well as non-invasive biomarkers of IBD, with much scope for future improvement and research.

## 1. Introduction

### 1.1. Inflammatory bowel disease and its diagnosis

Inflammatory bowel disease [IBD], the umbrella term for ulcerative colitis [UC] and Crohn’s disease [CD]—two chronic inflammatory gastrointestinal disorders of mixed environmental and genetic factor aetiology—has always been more prevalent in the Western world when compared with the East. Many epidemiological studies show an increase in incidence in IBD in Western populations in the second half of the 20th century, with a subsequent stabilising or possibly a small decrease in incidence in the same population.^1,2^ More recently, population-based cohort studies have shown an increase in prevalence of IBD in the East as well; and a global increase in rates of paediatric-onset IBD.^3,4^

These studies indicate that the global burden of IBD will increase in the coming years and also its associated morbidity and mortality—and both remain chronic relapsing and remitting disorders with no overt cure although chronic remission can be maintained.

The European Crohn’s and Colitis Organisation [ECCO] recommends clinicians use a multitude of tools, starting from history, examination, blood tests, stool tests, radiological imaging, and finally endoscopic visualisation of the colon or terminal ileum with or without histological analysis to diagnose UC or CD, rather than one single test.^5^ In recent years, faecal calprotectin has gained much traction as a relatively non-invasive marker of gut inflammation and is the newest biomarker in use for IBD diagnosis and disease monitoring—however, it is limited by its inability to differentiate IBD from other inflammatory disorders such as infectious gastroenteritis, false elevation with certain drugs, and the fact it is less recommended for use in patients over 60 years old.^6^

The main modality for IBD diagnosis remains endoscopy, and biopsy for histological assessment. However, colonoscopy is a finite, precious resource still recovering from the effects of the COVID-19 pandemic.^7^ In addition to this, IBD patients are complex and do not always present classically, exhibiting varying symptoms and disease courses when compared with other gastrointestinal disorders. ECCO recommends that a multitude of tests be used to gain an overall assessment of suspected IBD patients. Hence, another reliable biomarker for IBD would aid the diagnostic process for IBD patients.

Faecal immunochemical testing [FIT] has been widely used to triage patients for colonoscopy in population-based colorectal cancer screening programmes. However, for the diagnosis of IBD, FIT unfortunately diminishes in diagnostic accuracy. It is of low yield in detecting small bowel CD in the small bowel, is no better than faecal calprotectin for UC, and cannot technically distinguish between haemoglobin as a result of IBD or neoplasia.^8^ Volatile organic compounds [VOCs] may potentially serve the need for a novel, non-invasive biomarker of IBD.

### 1.2. Volatile organic compounds [VOCs] as biomarkers

Volatile organic compounds [VOCs] are carbon-based molecules that are gaseous at room temperature and may be emitted from the body as end products of metabolism such as breath, sweat, urine, and faeces. Disease processes in the body often involve alterations in metabolism and thus new production of VOCs or alteration in existing VOC patterns. Herein lies the principle for the potential for VOCs as biomarkers of organic gastrointestinal disease, a proposition which is gaining traction in modern research.^9^

#### 1.2.1 Volatile organic compounds [VOCs] sampling and measurement

Most research into VOCs as biomarkers of gastrointestinal disease has investigated three modalities for sampling from humans—breath, urine, and faeces. They are thought to be directly detectable in faeces or enter the systemic circulation and are then excreted from the body either via the kidneys as urine or through the lungs as breath.^9^

All three of these media are considered ‘non-invasive’ when compared with blood tests or endoscopy and liquid biopsy or tissue biopsy. However even among the three, urine is considered easier and more pleasant to sample than faeces and breath is even easier to give from a patient’s point of view.^10^ Therefore targeting VOCs as non-invasive biomarkers of IBD is advantageous as compared with other potential biomarkers such as methylated DNA or microRNAs^11,12^ and should be more acceptable to patients. This is essential when looking for a biomarker for potential use in disease screening and disease monitoring—it must be relatively cheap, non-invasive, and acceptable to patients, among other characteristics. VOC biomarkers can potentially satisfy all these criteria.

When it comes to VOC detection, broadly speaking there are two main divisions of technologies: metabolomic analytical methods and electronic noses.

##### 1.2.1.1. Metabolomic analytical methods

This subgroup describes a variety of chemical detection platforms such as gas chromatography mass spectrometry [GC-MS], nuclear magnetic resonance spectroscopy [NMR], and selected ion flow tube mass spectrometry [SIFT-MS], all of which have been used to detect specific VOC metabolites in clinical samples.

Gas chromatography mass spectrometry [GC-MS] involves the principle of chromatography—the separation of components in a mixture, based on their individual physical and chemical properties, and based on the amount of time they are mobile and stationary in different phases. This can be done with gases or liquids—gas chromatography or liquid chromatography. This can then be combined with mass spectrometry. The components are then placed into a vacuum and ionised, with the resulting ions further separated based on their mass to charge ratio, using electromagnetic fields. These ions are then detected and interpreted as signals or ‘peaks’, allowing the relative abundance of molecules within the concentration to be measured.^13^ This is a powerful and time-tested analytical technique which has been used in many studies for assessing VOCs as biomarkers.

In gas chromatography ion mobility spectrometry [GC-IMS], once molecules are separated they are introduced into an ion mobility spectrometer that ionises the molecules, which are then passed through a drift tube under the influence of an electric field, with different ions of different masses exhibiting different drift velocities. They then hit a plate and give off a time-dependent signal which can then be measured.^14^

In ion molecule reaction mass spectrometry [IMR-MS], once again a sample gas is ionised and introduced to reagent ions in a reaction chamber; after which they are put into the mass spectrometer. There is no performance of gas chromatography before these steps, and some advocate its ability to apply direct sample loading technology with possible reduced time of analysis.^15^

Selected ion flow tube mass spectrometry [SIFT-MS] is another powerful analytical technique that enables rapid and accurate measurement of VOCs. It involves insertion of a gas sample into a flow tube where it is mixed with selected reagent ions [most commonly H_3_0^+^, NO^+^, and O_2_^+^], and chemical reactions produce analyte ions which then go on to give signals of varying intensities that can be detected by a mass spectrometer further downstream.^16,17^ Over time, as technology has improved, the instruments used to carry out this technique have become smaller and more portable, and sensitivity of analysis has also improved. Many papers have used this technique to study VOCs from human samples.

Field asymmetric ion mobility spectrometry [FAIMS] is a tool that again involves ionisation of a gas sample, but it is then introduced into a narrow gap between two electrodes with application of a high-frequency asymmetrical waveform. This causes the ions to undergo cyclical motion and net drift in one direction based on the ions’ size, shape, and charge. At this point they can again be introduced into a mass spectrometer and undergo further separation before detection.^18^

These methods generally involve the use of complex machinery and software to quantify certain chemicals within the sample provided. One of the advantages of such approaches includes increased specificity to certain chemicals, allowing us to focus on certain metabolic pathways indicative of specific disease processes. They also give more detailed chemical information, and are highly reproducible with little evidence of the phenomenon of ‘sensor drift’ over time. However, the disadvantages include expensive operating and maintenance costs for machinery, the need for highly trained individuals, labour-intensive processes, and finally the long delay associated with results.^19^

##### 1.2.1.2. Electronic nose technology

In 1982, Persaud and Dodd constructed an electronic model of three gas sensors as transducers to mimic the discriminatory activity of the mammalian olfactory system for various odourants.^20^ This seminal work on the ‘artificial nose’ has formed the basic principle for the development of various electronic noses or ‘e-noses’ in the past 40 years. These remarkable devices identify overall ‘breathprints’ and are able to discriminate various aromas through varying stimulation of different gas sensors by volatile compounds. They do not, generally speaking, aim to identify quantities of individual compounds as with metabolomic analyses, and are negatively affected by sensor drift. However they are relatively small, cheap, and easy to use, with much less training required for operation. They also give relatively quick results.^21^

Electronic noses use a different variety of sensors in order to mimic mammalian olfactory systems and form a chemical reaction between the volatile organic compound and the sensor. Some of the types of sensors are as follows.

1. Metal oxide sensors—these sensors detect changes in electrical resistance in response to certain volatile organic compounds.^22^
2. Conducting polymer sensors—once again, these sensors change in resistance when exposed to certain vapours and volatile compounds.^23^
3. Quartz crystal microbalance/piezoelectric sensors—these devices have different oscillation frequencies when exposed to certain gaseous chemicals, which can be measured.^24^

Whereas most research into VOCs as biomarkers of gastrointestinal disease is oriented towards the diagnosis of upper and lower gastrointestinal cancer,^25,26^ there have also been case-control studies and cohort studies of faecal, breath, and urinary VOCs in inflammatory bowel disease. In this study, we aim to provide an up to date review of the available literature on studies of diagnostic accuracy of VOCs in inflammatory bowel disease, and to calculate pooled sensitivity and specificity data. We hypothesise that VOCs will display high levels of sensitivity and specificity for IBD.

## 2. Materials and Methods

A systematic review and a meta-analysis were conducted to capture and analyse the currently published data on the diagnostic accuracy of VOCs for the detection of IBD. This was carried out in accordance with the guidance from the Cochrane Handbook and has been reported in accordance with the Preferred Reporting Items for Systematic Reviews and Meta-Analyses guidelines.^27^

### 2.1. Search strategy

Literature search strategies were developed using medical subject headings [MeSH] and text words related to the research question. Then the search was carried out on the Medline, EMBASE, and Scopus databases by using various combinations of keywords and subject headings: ‘inflammatory bowel disease’, ‘volatile organic compounds’, ‘ulcerative colitis’, ‘Crohn’s disease’, and ‘volatolome’. There was no time limit or restriction placed on when the study must have been published by. To ensure maximum capture, we also scanned the reference lists of included studies and relevant reviews that had been identified via our search for potential studies.

### 2.2. Study selection

The inclusion criteria were as follows:

[1] prospective and retrospective comparative cohort studies, case-controlled studies, cross sectional studies, and randomised controlled trials;
[2] adult and paediatric studies of diagnostic accuracy of VOCs in confirmed IBD;
[3] published in English and available as full text in the medical database;
[4] IBD confirmed either through endoscopy or histology.

Only studies that gave sensitivity and specificity values for VOC analysis in the diagnosis of IBD were included in the meta-analysis.

The exclusion criteria were as follows:

[1] studies which did not have a control group;
[2] studies that were published as reviews;
[3] studies not published in English; abstracts and unpublished studies.

Two independent authors [AK and MA] screened the titles and abstracts of the papers to ensure they matched the inclusion criteria of our study sequentially. The screening was carried out in an independent manner, with ineligible studies excluded if study designs did not match the criteria for meta-analysis.

**Figure 1.**
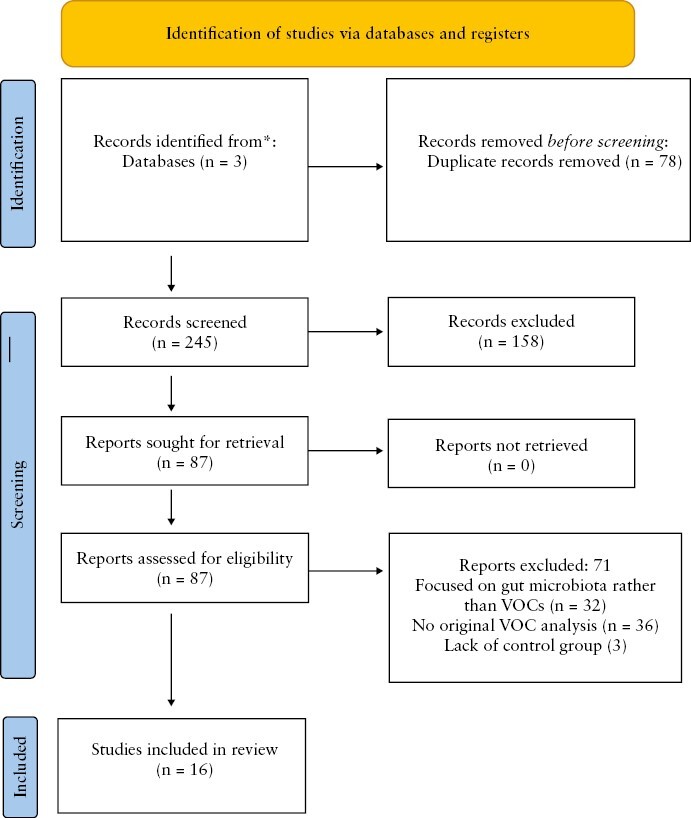
PRISMA Flow Diagram showing identification and exclusion of studies.

Where both authors agreed on study exclusion－‘congruent exclusion decisions’－this led to straight exclusion of the study. In the case of doubt over the validity of study inclusion－‘incongruent exclusion decisions’－a full-text review was undertaken, with SC acting as independent mediator in case of disagreement. The full flowchart of study selection is represented in the PRISMA diagram in Figure 1.

After searching three databases [MEDLINE, Embase, and Scopus] using the MeSH terms detailed above, a total of 323 papers were retrieved; 78 duplicate records were removed, leaving 245 abstracts to screen through. From abstract review, 158 papers were further excluded, leaving 87 papers. With institutional access we were able to get full texts for all 87 papers, and then a further 68 papers were excluded as they did not analyse VOCs. This left 16 papers that fulfilled the inclusion criteria for the systematic review. Of these 16 papers, 10 papers [with 11 cohorts] were suitable for meta-analysis.

### 2.3. Quality assessment and data selection

The QUADAS-2 tool was used to assess the quality of the included studies. This was undertaken by one reviewer and double-checked by the other. The studies were assessed against four domains—patient selection, index test, reference standard, and patient flow and timing; the risk of bias for each domain was grade as low, high, or unclear. Any disagreements between grades were resolved by discussion. Finally, publication bias was assessed using funnel plots and Deek’s regression-based method.^28^

### 2.4. Data synthesis and analysis

The following data were extracted from the papers: VOC results [true-positive—TP, false-positive—FP, true-negative—TN, false-negative—FN, sensitivity, specificity; cases, controls, analytical methods, sample matrix used, and comparator.

The R software was used for all statistical analyses.^29^ Where studies had reported only sensitivity and specificity, the TP, TN, FP, and FN were calculated, to produce 2 × 2 contingency tables. Bivariate analysis was performed using the package ‘mada’.^30^ Forest plots were created in order to illustrate sensitivity, specificity, and their corresponding confidence intervals. A summary receiver operating curve [SROC] with confidence regions for sensitivity and false-positive rate [1-specificity] was produced. The area under the curve was calculated to assess the overall accuracy. The heterogeneity was investigated through a visual distinction of forest plots, SROC, and correlation coefficient of sensitivity and specificity. Publication bias was evaluated through Deek’s regression test and funnel plot.

## 3. Results

### 3.1. Basic characteristics of studies

The results of the selection process are shown as a PRISMA diagram in Figure 1 with 16 studies^31–46^ extracted for systematic review and 10 [with 11 cohorts] suitable for meta-analysis. The basic characteristics of the included studies are summarised in Table 1.

**Table 1.** Basic characteristics of all studies included. Studies highlighted in bold were selected for meta-analysis.

Study [first author *et al.*]	Patient population	VOC medium	Analytical method	Study population	Sensitivity/specificity
Walton 2013^34^	Adult	Faecal	GC-MS	20 UC 22 CD 19 controls	Not given
Arasaradnam 2013^35^	Adult	Urine	e-nose/FAIMS	24 UC 24 CD 14 controls	Not given
**De Meij 2014** ^36^	**Paediatric**	**Faecal**	**e-nose**	**26 UC** **29 CD** **28 controls**	**94%; 94%**
Patel 2014^37^	Paediatric + Adult	Breath	SIFT-MS	11 UC 51 CD 55 controls	Not given
Hicks 2015^38^	Adult	Breath	SIFT-MS	20 UC 20 CD 18 controls	Not done for IBD Overall
**Arasaradnam 2016** ^39^	**Adult**	**Breath**	**FAIMS**	**29 UC** **25 CD** **22 controls**	**74%; 75%**
Ahmed 2016^40^	Adult	Faecal	GC-MS	100 UC 117 CD 109 controls	Not given
**Monasta 2017** ^41^	**Paediatric**	**Breath**	**IMR-MS**	**33 UC** **34 CD** **167 controls**	**96%; 69%**
Dryahina 2017^42^	Adult	Breath	SIFT-MS	54 UC 149 CD 14 controls	Not given
**Van Gaal 2017** ^43^	**Paediatric**	**Faecal**	**FAIMS**	**13 UC** **23 CD** **24 controls**	**79%; 78%**
**Bosch 2018** ^44^	**Paediatric**	**Faecal**	**FAIMS**	**2 UC** **15 CD** **25 controls**	**94%; 96%**
**Bosch 2018** ^45^	**Paediatric**	**Faecal**	**FAIMS**	**15 UC** **15 CD** **30 controls**	**93%; 97%**
**Tiele 2019** ^46^	**Adult**	**Breath**	**e-nose/GC-IMS**	**14 UC** **16 CD** **9 controls**	**67%; 89% [e-nose]** **87%; 89% [GCIMS]**
**El-Manouni el Hassani 2019** ^47^	**Paediatric**	**Faecal**	**GC-IMS**	**5 UC** **5 CD** **10 controls**	**70%; 90%**
**Bosch 2020** ^48^	**Adult**	**Faecal**	**GC-IMS**	**143 UC** **191 CD** **227 controls**	**97%; 92%**
**Bosch 2022** ^49^	**Adult**	**Faecal**	**e-nose**	**39 UC** **24 CD** **63 controls**	**78%; 59%**

CD, Crohn’s disease; UC, ulcerative colitis; IBD, inflammatory bowel disease.

Studies were eligible for meta-analysis if they gave values of sensitivity and specificity for the diagnosis of IBD as a whole versus controls, as this would allow us to generate TP, TN, FP, and FN values for meta-analysis. Six studies were not eligible for meta-analysis as they did not give sensitivity/specificity values for the diagnosis of IBD from their study, some giving other measures such as area under the curve [AUC] or comparing UC and CD individually with controls.

In terms of basic study characteristics, there was a mix of 10 adult and six paediatric studies. Faecal samples were the most studied VOC medium, with nine papers focusing on them; six papers carried out breath analysis and only one study analysed urine samples.

With regard to analytical techniques—only four studies used electronic noses, with 12 studies using metabolomic methods such as GC-MS, SIFT-MS, FAIMS, gas chromatography ion mobility spectrometry [GC-IMS], and ion molecule reaction mass spectrometry [IMR-MS]. Quality assessment of the studies was carried out with the use of the QUADAS-2 tool [as detailed above] and this is shown in Table 2.

**Table 2.** Assessment of bias in studies included in meta-analysis according to the QUADAS-2 tool.

Study [first author *et al*.]	Patient selection	Index test	Reference standard	Flow and timing
De Meij 2014^36^	Unclear	Unclear	Low	Low
Arasaradnam 2016^39^	High	Low	Low	Low
Monasta 2017^41^	High	Unclear	Low	Low
Van Gaal 2017^43^	Low	Low	Low	Low
Bosch 2018^44^	High	High	Low	Low
Bosch 2018^45^	Low	High	Low	Low
Tiele 2019^46^	Unclear	High	Unclear	Unclear
El-Manouni el Hassani 2019^47^	High	Low	Low	Low
Bosch 2020^48^	Unclear	Low	Unclear	Unclear
Bosch 2022^49^	High	High	Low	Low

**Figure 2.**
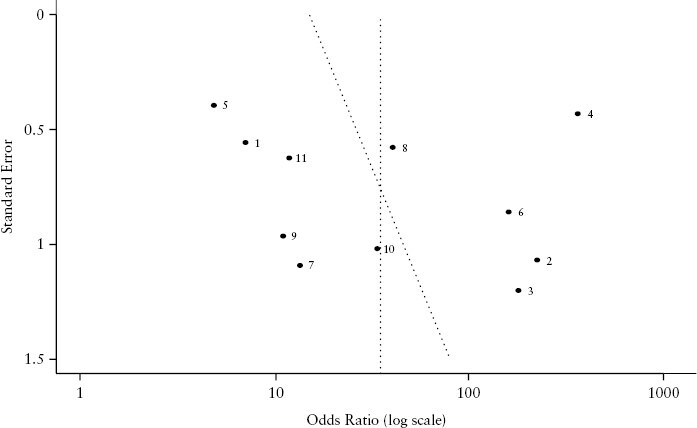
Deeks’ Regression plot with test for Funnel Plot Asymmetry: p = 0.7808 – implies a lack of publication bias.

The major risk of bias was in the ‘Patient Selection’ and ‘Index Test’ domains, with five studies showing high risk of bias in each of these domains. This was determined through the methodology of the papers, with studies often selecting known IBD cases and controls rather than consecutively referred patients. However, studies generally showed a low risk of bias with regards to the ‘Reference standard’ and ‘Flow and Timing’ domains. Furthermore, Deek’s regression test showed a *p*-value of 0.78, which suggests absence of publication bias; this is shown in Figure 2. There was a mix of adult and paediatric studies included in the meta-analysis.

The analytical methods and sample medium used for the detection of VOC varied across the studies. The correlation coefficient of sensitivity and specificity was 0.31, which suggests presence of heterogeneity among the studies included in the meta-analysis. Meta-regression analysis was performed to explore the heterogeneity further. The covariates included were sample medium and analytical techniques used. The diagnostic accuracy of VOCs in distinguishing UC from CD could not be included in the regression analysis, as the studies did not report separate control groups for UC and CD. Neither the sample medium nor the analytical techniques reached statistical significance in the regression analysis. However, this should be interpreted with caution as the number of studies and overall population size were small; and therefore we have not drawn any meaningful conclusions from this. Overall however, many of the studies exhibited high levels of diagnostic accuracy for IBD with high sensitivity and specificity, and this is reflected in our meta-analysis.

### 3.1. The diagnostic accuracy of VOC for the detection of IBD

Meta-analysis was performed on 10 studies [11 study cohorts]. The study by Tiele *et al.*^43^ had two separate study cohorts. The studies targeted patients by taking samples prior to diagnosis and before medical treatment was started. Some studies did look at disease monitoring, which will be explored later. When pooled, the studies overall contributed 696 cases of IBD [321 UC patients; 375 CD patients] and 605 control patients for meta-analysis.

**Figure 3.**
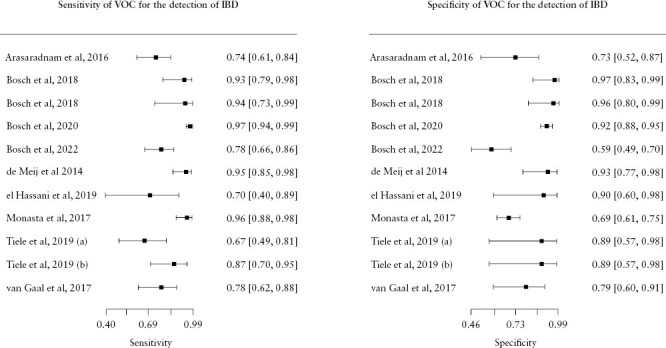
Forest Plot of studies of sensitivity and specificity of VOC for the detection of IBD.

**Figure 4.**
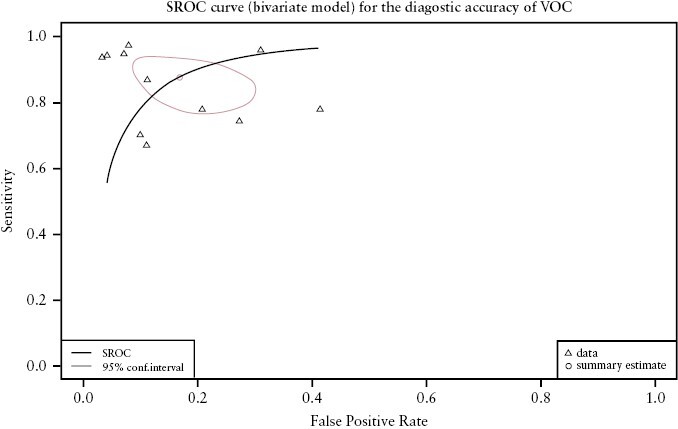
Summary receiver operating characteristic (SROC) curve for the diagnostic accuracy of VOCs in IBD. Area under the curve is 0.92.

Figure 3 illustrates forest plots showing sensitivity, specificity, and their corresponding confidence intervals for the included studies. Figure 4 represents the summary receiver operating characteristic [SROC] curve analysis for VOCs. The pooled sensitivity and specificity of VOCs for the detection of IBD was 0.87 [95% CI, 0.79 - 0.92] and 0.83 [95% CI, 0.73 - 0.90], respectively. The AUC of the SROC was 0.92.

These results are positive, with a pooled sensitivity and specificity of 87% and 83%, respectively, across 696 IBD patients versus 605 controls; and seem to affirm our a priori hypothesis that VOCs can serve as biomarkers for IBD.

### 3.2. Systematic review of studies not included in the meta-analysis

Six studies were not eligible for meta-analysis but still provided valuable insight into the research question.

Walton *et al*.^31^ used GC-MS to analyse faecal samples from patients with UC, CD, irritable bowel syndrome [IBS], and healthy control patients. They found statistically significantly elevated concentrations of ester and alcohol derivates of short chain fatty acids and indole in Crohn’s disease when compared with controls. They also showed that after treatment, many of these VOCs reduced in concentration, alluding to a ‘normalisation’ of the VOC profile. The main limitation of this study was the lack of a diagnostic model for IBD with any calculation of diagnostic accuracy, and this perhaps reflects its status as one of the earliest studies on VOCs in IBD, published in 2013.

Arasaradnam *et al.*^32^ was the only study that looked at VOCs in urine samples and used both FAIMS and an e-nose to show that IBD could be discriminated from healthy controls with an accuracy of 75%, through a Fisher’s discriminant analysis.

Patel *et al*.^34^ used SIFT-MS to classify breath samples from IBD vs control patients, with an area under the curve of 0.96 [95% CI, 0.93 - 0.99], but could not demonstrate differences between UC and CD; or any significant correlation with disease activity.

Hicks *et al.*^35^ created a statistical model to discriminate paediatric IBD breath samples from healthy paediatric control breath samples and UC from CD. UC was differentiated from controls with a sensitivity and specificity of 94% and 94%, respectively, and UC from controls with a sensitivity and specificity of 91% and 94%, respectively. CD was differentiated from UC with a sensitivity and specificity of 89% and 90%, respectively. Furthermore, this paper identified statistically significant differences in dimethyl sulphide, hydrogen sulphide, and nonanal in CD versus healthy controls. They were unable to find statistically significant differences in individual compounds for UC vs healthy controls.

Ahmed *et al*.^37^ analysed faecal VOC samples by GC-MS and found separation of active CD patients from inactive disease and healthy controls, but could not demonstrate separation between active UC, inactive UC, and healthy controls.

Finally, Dryahina *et al.*^39^ carried out SIFT-MS analyis on breath samples of IBD and healthy control adult patients. They noted a greater abundance of pentane, hydrogen sulphide, acetic acid, propanoic acid, and butanoic acid in IBD patients vs healthy controls. They postulated that this could be due to bacterial overgrowth in these diseases. Again, their limitation was in no calculation of diagnostic accuracy.

### 3.3. The diagnostic accuracy of VOCs for differentiating UC from CD

A few studies tried to answer the question of whether VOC analysis can differentiate UC from CD, since we know that the pathophysiology [and therefore the underlying metabolic processes] varies between the two diseases. Furthermore, in clinical practice this can be a difficult undertaking and is mainly based on differentiation through varying endoscopic and histological features. As explained above, however, we were unable to carry out meta-analysis since the studies did not report separate control groups for each subgroup of patients with either UC or CD. Nevertheless, we qualitatively reviewed the six studies that reported VOC patterns in UC versus CD. The results were mixed.

Three studies reported positively on the discriminatory ability of VOC analysis between UC and CD. Hicks *et al.*^35^ used their model to differentiate 18 CD cases from 20 UC cases, with a sensitivity and specificity of 89% and 90%, respectively. Similarly, Arasaradnam *et al.*^36^ used FAIMS in adult samples to differentiate 29 UC cases from 25 CD cases, with a sensitivitiy and specificity of 67% and 67%, respectively. Finally, Tiele *et al*.^43^ reported differentation of 16 UC cases from 14 CD cases, with a sensitivity and specificity of 71% and 88%, respectively [*p* <0.0001].

However, Van Gaal’s cohort^40^ included 13 UC patients and 23 CD patients. They quoted a sensitivity and specificity of 65% and 62%. respectively, for differentiating between the two groups, and found this was not statistically significant [*p* = 0.1] and concluded that VOC analysis could not confidently differentiate between UC and CD. Similarly Bosch *et al*.,^42^ in their study in 2018, could not distinguish between 15 UC and 15 CD cases on the basis of faecal VOC profiles. Finally, Bosch *et al*.^45^ in 2020 reported that faecal VOC profiles showed differences but diagnostic accuracy was very low, with sensitivity of 17% and a negative predictive value of 36%.

Therefore at present there can be no firm conclusion on the accuracy of VOCs to discriminate between UC and CD.

### 3.4. The diagnostic accuracy of VOCs in IBD disease monitoring

One important utility for a biomarker lies in disease monitoring, with the ability to predict relapses into disease from a remission state. This is especially relevant in chronic, remitting, relapsing conditions like inflammatory bowel disease. Currently the most frequently used biomarkers for IBD disease monitoring are serum C-reactive protein [CRP] and faecal calprotectin.^47^

Five studies^31–33,37,41^ were able to show a clear difference in VOC profiles after patients went into remission from active disease, although only Walton *et al*.^31^ described the treatment regimen given. This was steroids and 5-aminosalicylic acid for patients with UC, and elemental diet for patients with CD. These studies did not follow the patients up for longer periods of time or comment on any relapses.

Only one study, Bosch *et al*.,^46^ looked at the utility of faecal VOCs in relapses over a period of time. Interestingly, they were able to show a difference in faecal VOC profiles between patients preceding relapses versus those who remained in remission, with an area under the curve of 0.75 [*p* <0.01]. Furthermore, this difference in VOC profile even preceded faecal calprotectin changes, and it was theorised that VOCs could potentially predict disease relapses even earlier, thus allowing earlier treatment and reduced complications and hospital admissions.

## 4. Discussion

In the ongoing search for a non-invasive biomarker for IBD, our review into VOCs raises several interesting points.

Our study shows that this is an exciting time for VOC research, with all manuscripts being recent, ie, published in the past 10 years. There has also been a clear evolution in the studies, with earlier manuscripts often published as ‘proof of principle’ studies and later studies building on the methodology of these earlier studies. This is apparent when looking at Table 1—all studies included in the past 5 years have come up with statistical models on their individual VOC analyses to calculate diagnostic accuracy in terms of sensitivity and specificity; which did not happen in the earlier studies.

There have been two recent reviews—van Malderen in 2020^48^ and Vernia in 2021^9^—that also looked at the use of VOCs as diagnostic biomarkers for IBD, and both concluded that VOCs hold significant promise in this respect. However our study goes a step further, with nested meta-analysis which is, to our knowledge, the first of its kind in this field and provides a contemporary quantitative portrayal of where we are with regards to VOC research for biomarkers in IBD.

With a pooled sensitivity and specificity of 87% and 83%, respectively, across 696 IBD patients vs 605 controls, it is clear that VOCs show much promise as non-invasive biomarkers of IBD. They do not require blood samples and, as our studies show, we are getting more used to collecting and transporting VOCs through various devices such as thermal desorption tubes, and to analysing them with instruments such as e-noses, which we would expect only to increase in number.

There are some limitations to our study. First, these are all case-control studies and we do not have any prospective, randomised studies with clear blinding procedures followed. This is reflected in our analysis of the studies by the QUADAS-2 tool in Table 2—where many studies are at risk of bias in the patient selection and index test domains.

Second, there is heterogeneity among our studies. There does not appear to be any consensus on which is the best VOC medium to analyse—urine, faeces, or breath—although it is interesting that most studies look at breath or faeces rather than the one paper we found that looked at urine. Breath is easier to sample than urine or faeces from a patient point of view, and urine would be easier than faeces to sample. There is also no consensus on the best analytical method—whether it be through the use of more complex metabolomic techniques or electronic noses. It is, however, clear and interesting to note that we are yet to discover one or two compounds that are present in disease states compared with the healthy physiological state, for cancer or inflammation. This suggests that the overall picture/variable composition of VOCs is more pertinent, and thus metal oxide-based technology may be more appropriate going forward in this disease group. It also carries the advantage of being operable with minimal training. Furthermore, the fact that VOCs of all media show correlation with disease states means that there are more potential future diagnostic targets, rather than being limited to a particular sample type.

Whereas the studies in our meta-analysis appeared to differentiate IBD from controls very well, it appears that differentiating UC from CD is more difficult. Nevertheless, the VOC profile of CD patients appears more distinct than UC or healthy controls, as evidenced by Hicks *et al.*^35^ and Ahmed *et al*.^37^ We propose that this may be because CD is a transmural disease with greater perturbation of metabolic pathways when compared with UC.

The influence of confounding factors, such as age, gender, body mass index [BMI], diet, drugs, smoking and metabolic comorbidities, into VOC abundance^49^ is another facet of this research area that needs further clarification and potential adjustment when using VOCs as biomarkers. For example, smokers would introduce certain exogenous VOCs into breath samples looking for disease; once these are known they could be accounted for, by adjustments to probabilistic neural networks generated from e-nose outputs.

Currently faecal calprotectin [FCP] and CRP are the most widely used non-invasive biomarkers for IBD and are often used in first diagnosis and disease monitoring. Calprotectin is a protein released by neutrophils as part of inflammation in the bowel, which is stable in faeces and thus can be measured by laboratory methods.^50^ CRP is an acute-phase protein that is released by the liver in response to macrophages and T cells as part of the immune response to systemic inflammation.^47^ Therefore these two tests are together surrogate markers of both local bowel and systemic inflammation. A raised CRP is not specific to gastrointestinal inflammation; and a raised FCP is not specific to inflammatory bowel disease.

We propose that VOCs could be more attractive than current biomarkers.

First, the potential for breath and urine samples to guide management could increase patient compliance and also lay the foundation for future point of care testing. These samples are easier to take from patients than blood or stool samples, particularly when it comes to the paediatric population of IBD which is set to increase in number. Delivered at scale, it is comparative in cost with faecal calprotectin.

Furthermore—rather than purely acting as surrogate markers of inflammation—VOCs could give more information and distinguish between certain diseases, for example between infective gastroenteritis, UC, CD, or cancer, as VOC patterns are disparate based on the underlying disease.^51–53^ There is more scope to evolve and observe different patterns of recognition.

It is noteworthy that the use of VOCs as a biomarker in IBD could be influenced by several factors. For example, faecal calprotectin is stable in stool for days at room temperature and therefore does not require specialised collection equipment or cold storage with defrosting later. This introduces additional steps of work and potential for variation or error, and standardisation of measurement techniques is required.^54^ Individual metabolic and environmental factors, such as renal failure or exogenous drugs, can lead to variation in VOCs when compared with current biomarkers and this needs to be considered.^55^ However, there are emerging data to show distinction between, for example, irritable bowel syndrome and bile acid diarrhoea^56^ and different cancer types of differing cell origin.^57^ Studies so far have also not elucidated the variations in VOCs for subsets of disease, such as acute severe UC or in fibrotic CD strictures with pre-stenotic dilatation, but faecal calprotectin also cannot distinguish between these subsets.

However, we anticipate that these are all challenges that may be overcome in future—with impending studies involving larger numbers of patients over multiple centres and blinding of those collecting samples as well as of those analysing the samples, to reduce any potential biases. There should also be more focus on differentiating UC from CD; and on using these compounds as markers of disease activity or remission. Research into VOCs as biomarkers is therefore at a relatively early but exciting stage, with large potential for growth and benefit to patients.

In conclusion, VOCs are easily sampled, low cost, and hold very good diagnostic accuracy for IBD. Going forward, larger, multicentre, prospective studies are required to formulate the best methods of sampling, analysing, and interpreting VOCs as biomarkers of IBD.

The data that support the findings of this study are freely available from the corresponding author upon reasonable request.
